# Unravelling hidden hearing loss

**DOI:** 10.7554/eLife.104936

**Published:** 2024-12-05

**Authors:** Emmanuel Ponsot

**Affiliations:** 1 Science & Technology of Music and Sound Laboratory, IRCAM/CNRS/Sorbonne Université Paris France

**Keywords:** hidden hearing loss, cochlear neurodegeneration, cochlear synaptopathy, hair cells, dementia, envelope following response, Human

## Abstract

Damage to the synapses connecting hair cells to the auditory nerve leads to undetected hearing impairments.

**Related research article** Zink ME, Zhen L, McHaney JR, Klara J, Yurasits K, Cancel V, Flemm O, Mitchell C, Datta J, Chandrasekaran B, Parthasarathy A. 2024. Increased listening effort and cochlear neural degeneration underlie behavioral deficits in speech perception in noise in normal hearing middle-aged adults. *eLife* **13**:RP102823. doi: 10.7554/eLife.102823.

Age-related neural changes start to occur in the brain during middle age. For example, hearing loss can begin at around 40 years of age. However, many middle-aged adults who struggle to understand speech in noisy environments have clinically normal hearing tests. This 'hidden hearing loss' could be due to faults in the transmission of sound signals to the brain that are not detectable with conventional tests (Liberman, 2024).

Loud noises, typical aging processes or ototoxic drugs can cause irreversible damage to the synapses between the inner hair cells of the cochlea – the sensory receptors in the inner ear – and the auditory nerve fibers that transmit sound information from the cochlea to the upper auditory processing stages in the brain (Kujawa and Liberman, 2009). This damage, known as cochlear synaptopathy, is usually permanent and can lead to delayed degeneration of the cochlear nerve. However, research in mice has shown that sound detection thresholds can return to normal a few days after injury. The precise impact of cochlear synaptopathy on human hearing and how it might impact our ability to understand sounds, such as speech in noisy environments, is still under debate (for a recent review, see Liu et al., 2024).

One of the reasons for this is that cochlear synaptopathy can only be studied indirectly in humans, using non-invasive techniques (Bramhall et al., 2019). Since these measures only detect correlation, efforts have been made to verify findings using cross-species studies and computational models (Bharadwaj et al., 2022; Encina-Llamas et al., 2019).

It is also challenging to evaluate the impact of cochlear synaptopathy on real-world speech perception. Some studies using simplified tasks, such as distinguishing digits or vowels, suggest a link between loss or dysfunction of auditory nerve fibers and individuals experiencing hearing difficulties. However, such tasks may oversimplify the complex nature of natural speech perception, potentially underestimating the role of the brain. Meanwhile, in more complex speech-in-noise tasks, commonly used indicators often cannot predict speech intelligibility scores as effectively as cognitive or attentional measures do (Kamerer et al., 2019; DiNino et al., 2022; Gómez-Álvarez et al., 2023). Now, in eLife, Aravindakshan Parthasarathy and colleagues – including Maggie Zink and Leslie Zhen as joint first authors – report new ways to overcome these obstacles (Zink et al., 2024).

To tackle the first challenge, the team – which is based at the University of Pittsburgh and Virginia Polytechnic Institute and State University – employed a cross-species approach, studying young and middle-aged humans as well as gerbils, which have the same hearing frequency range as humans (Figure 1A). Using electrodes placed on the earlobe and on the top of the forehead, Zink et al. measured the envelope following response (EFR), reflecting the ability of neurons to track rapid changes in stimulus envelope. This measurement, which can act as a proxy to detect cochlear synaptopathy, revealed a comparable decline in neuronal activity in both species from younger to middle-aged groups (Figure 1B).

**Figure 1.**
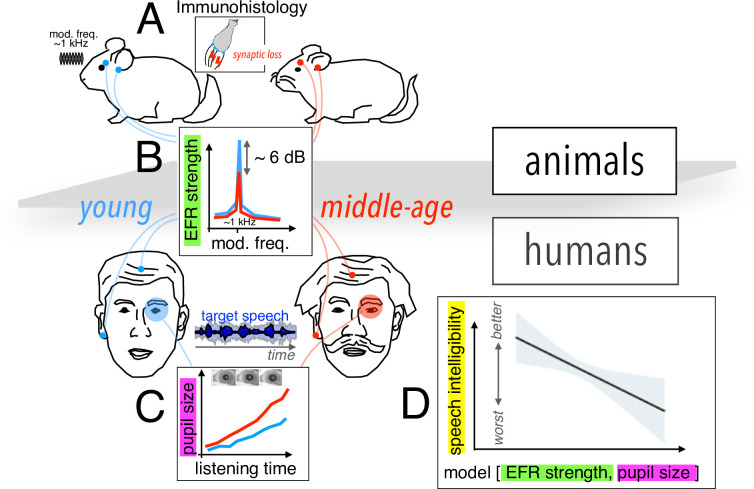
Assessing cochlear synaptopathy in humans and rodents. (**A**) Zink et al. used a cross-species approach to assess hidden hearing loss in humans and gerbils. Immunohistology in gerbils confirmed these animals suffered from cochlear synaptopathy. (**B**) A reduction in envelope following response (EFR) strength was found between middle-aged (red) and younger (blue) humans, and gerbils. (**C**) Larger pupil size was found in middle-aged humans decoding speech in noise. The magnitude of the ectroencephalographic-based assessment of sound envelope encoding (EEG) and pupil size were both significant predictors of speech intelligibility differences beyond hearing thresholds (**D**), and as such constitute the hidden dimensions of hearing deficits.

Further histology studies in gerbils counting the average number of remaining auditory nerve fibers per inner hair cell confirmed that these animals did indeed suffer from cochlear synaptopathy, highlighting a potential association between lower EFR magnitude and cochlear synaptopathy in humans. A computational auditory model showed that the size of the EFR decrease with age observed by Zink et al. is consistent with the proportional loss of synapses for these groups measured in previous histological and analytical studies (Wu et al., 2019; Zilany et al., 2014).

To address the second challenge, Zink et al. assessed speech-in-noise perception using a custom-designed task of intermediate complexity, while simultaneously using pupillometry to monitor pupil size, which is thought to be a proxy of listening effort (i.e., for the level of cognitive resources required by the brain to process the incoming neural signal; Figure 1C and D). Their results revealed an increased listening effort in middle-aged test subjects, even though intelligibility scores remained the same throughout the test. Crucially, a statistical model including predictors for both cochlear synaptopathy and listening effort showed that each factor contributed significantly to the speech-in-noise difficulties in the human cohort (Figure 1E).

In conclusion, while Zink et al. do not establish causation, their work represents a valuable step in understanding how early cochlear synaptopathy in middle-aged adults may contribute to speech-in-noise deficits. It also suggests that cognitive and attentional factors may play an even larger role than previously expected.

Yet, our understanding of how cochlear synaptopathy distorts neural coding and perception of sound in humans is still overly simplistic, and many questions remain unanswered. For example, little is known regarding how central processing stages of the brain are impacted and potentially compensate impaired peripheral sound transduction (Resnik and Polley, 2021). Moreover, it remains unclear which specific sound features’ encoding are distorted by cochlear synaptopathy and finally lead to degraded perceptual capacities, as many behavioral studies in animals with drug-induced cochlear synaptopathy show no evidence of impairment (Henry et al., 2024).

Middle age is a significant period during which age-related changes start to occur in the brain that could shed light on potential neurodegenerative conditions later in life, hearing loss being one of them. Further research is needed before a behavioral marker of cochlear synaptopathy becomes available at the clinic: a first step will be to refine current test proxies and to develop sensitive neural markers that could help identify the early onset of conditions such as dementia.
